# Physico-Chemical Characterization of Amino Acid-Based Deep Eutectic Solvents

**DOI:** 10.3390/molecules30040818

**Published:** 2025-02-10

**Authors:** Saffron J. Bryant, Gary Bryant, Calum J. Drummond, Tamar L. Greaves

**Affiliations:** School of Science, STEM College, RMIT University, Melbourne, VIC 3000, Australia; gary.bryant@rmit.edu.au (G.B.); calum.drummond@rmit.edu.au (C.J.D.); tamar.greaves@rmit.edu.au (T.L.G.)

**Keywords:** deep eutectic solvents, neoteric solvents, amino acid-based deep eutectic solvents, DES

## Abstract

Deep eutectic solvents are an exciting class of designer solvents that are increasingly gaining popularity. Deep eutectic solvents based on amino acids are particularly interesting for biomedical applications due to their potential low toxicity. However, very few have been reported to date, and only one of these has been comprehensively studied, made from a combination of proline and glycerol. Here, we report for the first time a systematic investigation into amino acid-based deep eutectic solvents, with a particular focus on the structural features of amino acids that promote eutectic formation and their influence on viscosity, refractive index, surface tension and thermal behavior. Of the 22 amino acids (and related compounds) examined, only 3 (lysine, arginine and, as previously reported, proline) formed stable homogenous liquids in combination with glycerol or ethylene glycol. For these mixtures, it was found that the second component (glycerol or ethylene glycol) had a much more significant influence on the physical properties than the identity of the amino acid. Most significantly, it was found that far fewer amino acids readily formed deep eutectic solvents than has been generally assumed. This is the first work to systematically characterize deep eutectic solvents based on amino acids and, as such, paves the way for future biomedical applications of these solvents.

## 1. Introduction

The specific definition of deep eutectic solvents (DESs) has been the cause of debate in the literature since their original description in 2003 [1]. They are often incorrectly classed as a subset of ionic liquids, or the DES label is given to any mixture of two or more compounds, both of which are incorrect [2]. More often, DESs are associated with hydrogen bonding, although this did not form part of the original definition which instead involved Lewis or Brønsted acids and bases [2]. In general, DESs are mixtures of components that have a melting point which is significantly below that of the individual components, and the true *deep* eutectic solvent is the ratio with the lowest eutectic point [3]. Abranches and Coutinho go into greater depth about these issues but suggest that there is no significance to the ratio of components and that mixtures should be used at whatever ratio makes them most useful [2]. While we agree that mixtures should be tuned in whatever way makes them most appropriate to desired applications, we feel there is value in reporting the mixture with the greatest melting point suppression (that is, the *deepest* eutectic solvent). This is a fundamental property of these solvents and provides information about molecular interactions.

Much like the issues surrounding the definition of DESs, inconsistencies exist in the characterization and reporting of properties of these solvents, even for the most basic properties such as melting point [4]. This is further complicated by the significant influence that the presence of even small quantities of water can have on the physical properties of these solvents [3,5].

Despite these problems, DES-like solvents have found applications in a range of fields including cryopreservation [6,7,8], antimicrobials [9,10], self-assembly media [11,12,13], lubrication [14] and catalysis [15]. Many emerging fields of interest involve biomedical applications such as drug delivery which necessitates biocompatible or non-toxic DESs [16,17]. For this reason, there has been an increasing focus on DESs composed of bio-derived components, such as amino acids. Many papers have reported multi-component amino acid-based DESs; however, many of these relied on extensive addition of water [18] or did not adequately describe the synthesis method so have not been reproduced [19]. One group reported a large number of DESs containing amino acids; however, the water content was either not reported or, where it was reported, often exceeded 15 wt%. In the absence of water, the majority of these combinations have been found to either not mix, or to form solids (see Supplementary Information) [20]. Despite this, these combinations appear repeatedly in the literature and in reviews as established DESs. Similarly to the evolution of ionic liquid definitions, it is reasonable to include mixtures with high water contents within the DES umbrella term, but we note that the water content must be emphasized and reported for repeatability.

Furthermore, the influence of water on physical properties must be properly studied and acknowledged. This is even more important given the hygroscopic nature of many DESs. For example, atmospheric exposure for just 48 h caused choline chloride/urea to absorb 5.5 wt% water, which resulted in a 15 °C decrease in melting point [5]. The impact of water on DESs is discussed at length in previous reviews [4,21] and research papers [3,22]. It is unsurprising that water can so strongly influence DESs as hydrogen bonding often forms such a key part of the solvent nanostructure. Water can be incorporated into this nanostructure, perhaps altering it even at low concentrations. However, the impact of water on DES properties will vary depending on the components of the DES [3]. For these reasons, water cannot be arbitrarily added to mixtures and expected to have no effect.

Some groups have focused on DESs utilizing acids such as lactic or acetic acid [23], but these compounds have been shown to be cytotoxic and therefore unsuitable for biomedical applications [24].

Our group recently published an extensive characterization of proline-based DESs utilizing glycerol or ethylene glycol as the hydrogen bond donor (HBD) component and found that, in both cases, a liquid with a significantly depressed melting point was formed [3]. We have been unable to find similarly rigorous investigations of other amino acid-based DESs in the literature. Therefore, this paper explores combinations of amino acids that use glycerol and ethylene glycol as the second component as these are already in use in biomedical applications [6,25,26,27]. While there are over 500 amino acids in nature, we will focus on those that are incorporated into proteins. Here, we report on the formation of homogenous mixtures and characterize the thermal behavior, surface tension, viscosity and refractive index. This paper demonstrates that far fewer amino acids form homogenous liquids with glycerol and ethylene glycol than has been suggested by the existing literature. These results highlight the novelty of our rigorous, systematic approach to DES synthesis and characterization. In particular, we demonstrate the following: (a) many amino acid DESs currently reported in the literature do not exist; (b) the HBD component has a more significant influence on the physical properties of these DESs than the amino acid; and (c) the physical properties of these novel DESs (such as broad thermal stability) make them promising for a range of applications, particularly biomedical.

## 2. Results and Discussion

### 2.1. Formation of Homogenous Liquids

As described in the materials and methods section, only 14 of the 52 combinations tested formed homogenous mixtures, and even the addition of water over 50 wt% did not have a significant effect in most cases. The only mixtures that formed homogenous liquids at low water contents (<2 wt%) were lysine and arginine (and as previously reported, proline [3]). This is different to the previous literature which reported a homogenous liquid formed by alanine–glycerol, glycine–glycerol, histidine–glycerol and threonine–glycerol at a 1:3 ratio of amino acid to glycerol [19]. That paper did not describe the method of synthesis in detail; however, it is unlikely to be a simple case of residual water, given the results described here. This discrepancy in results may be due to inadequate observation at room temperature (some of these mixtures may be homogenous at very elevated temperatures), impurities in the precursors, errors in calculating or reporting the ratio of amino acid to glycerol, and other methodological steps that may have altered the chemical identity of the components. Given that these so-called DESs have not been reproduced by other groups, we suggest that they do not in fact form homogenous liquids at room temperature and that rather, the results presented herein are more indicative of their true mixing behavior.

Table 1 shows the water contents of the mixtures that were taken forward for testing in this study. As described in the Materials and Methods section, those mixtures that required 50 wt% or more water to form a homogenous mixture were not examined further. It is apparent from Table 1 that all of the lysine-containing mixtures readily formed homogenous DESs with minimal water present. However, even with extensive drying using a GenVac (SP Scientific, Warminster, PA, USA, EZ-2 4.0), the water content in mixtures of lysine and glycerol could not be reduced below the values in Table 1. The mixtures with ethylene glycol were not subjected to further drying steps due to concerns about evaporation of the ethylene glycol, which has been observed in previous studies [28]. Mixtures of arginine with glycerol at 1:4.5, and at both ratios with ethylene glycol, formed homogenous liquids at the lowest water content. However, the arginine–glycerol ratio of 1:3 required 10 wt% water, suggesting this is further from the eutectic point. Interestingly, serine and glycine only formed homogenous liquids with large proportions of water (>20 wt%) present, and even then serine–EG and glycine–Gly crystallized after a few days at room temperature.

### 2.2. Thermal Behavior

**Figure 1:**
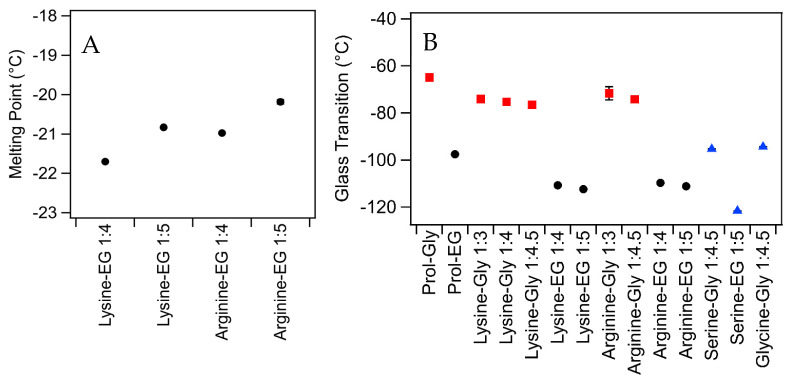
(**A**) Melting point and (**B**) glass transition temperatures of mixtures. Data for proline-based samples were taken from a previous publication [3]. Glycerol mixtures are shown as red squares, and ethylene glycol mixtures are shown as black circles. The blue triangles show samples that had >20 wt% water. Error bars are based on standard deviation of triplicate measurements and, in many cases, are smaller than the symbols.

Only four of the mixtures exhibited melting points (lysine and arginine in combination with ethylene glycol), and these are shown in Figure 1. Such melting behavior was not observed for ethylene glycol in combination with proline or serine. It must also be noted that the DSC thermograms did not show a simple freezing/melting behavior for these solvents, but rather a complicated series of recrystallisation and melting events during the warming cycle (Figure S1). A similar behavior has previously been observed for choline chloride–ethylene glycol mixtures, although the crystal structures of these phases are still under exploration [3]. Ethylene glycol on its own has a melting point of −12.7 °C which is higher than the melting points observed for mixtures with amino acids, suggesting that the presence of the amino acids suppresses melting [29]. The melting point of the lysine mixtures was lower than the equivalent arginine-based mixtures, suggesting that the additional nitrogen groups on arginine promote crystallization.

None of the glycerol-based mixtures exhibited melting, which is consistent with previous studies [3]. However, it must be noted that a small freezing peak was observed during the cooling runs of lysine and arginine in combination with glycerol (Figure S2). Given that there was no melting observed in DSC, and that none of these mixtures exhibited crystallization after storage for >8 weeks at room temperature (~22 °C), it is unclear what this small peak may be attributed to.

All of the liquid mixtures tested exhibited glass transitions (Figure 1B). The glass transition of glycerol-based mixtures was higher than that of equivalent ethylene glycol-based mixtures, consistent with the known glass-forming properties of glycerol [30]. Glycerol on its own has been shown to have a glass transition at −83 °C using a similar DSC protocol, which is slightly below that of the glycerol-containing mixtures examined here [31]. There was no clear link between glass transition temperature and amino acid, except that the glycine and serine mixtures had lower glass temperatures than the others. This may be attributed to the easier diffusion of small molecules, which delays the formation of an amorphous glass structure until lower temperatures, or the particularly high water content in these samples.

In all cases, the melting point and glass transition temperatures of these mixtures were lower than that of the most-studied DES (choline chloride–urea (1:2)) which has a melting point of ~25 °C and a glass transition temperature above −60 °C [3]. This suggests that these DESs may be more suitable for low-temperature applications where there is a risk of choline chloride–urea freezing.

As discussed above, the definition of a *deep* eutectic solvent has been debated extensively in the literature. A recent paper by Like et al. proposed a possible definition: that the negative molar excess Gibbs energy of the eutectic must be at least one-third that of the background thermal energy per mole (Equation (1)) [32],

(1) $$\frac{g^{e}}{RT_{e}}\leq -\frac{1}{3}$$

where *g^e^* is the molar excess Gibbs energy value, *R* is the universal gas constant (8.31 J/mol·K), and *T_e_* is the melting point of the mixture in K. This equation provides a measure of how ‘deeply’ the melting point is depressed relative to an ideal mixture (which would equal 0). A positive value would mean that the mixture has a melting temperature higher than that predicted for an ideal mixture, and a negative value means the melting temperature is lower than the ideal. While the choice of one-third is arbitrary, it does provide a clear cutoff to define deeply eutectic mixtures [3,33].

Calculation of the molar excess Gibbs energy requires knowledge of both the melting point and enthalpy of mixing of the components of the mixture. In the original publication by Like et al., they utilized the decomposition data for many components as there was no ‘melting’ point [32]. This is also the case for the amino acids studied here. Therefore, theoretical values for melting temperatures of the amino acids were used based on the Joback method which estimates physical properties based on contributions from molecular functional groups [34]. While not 100% accurate, this method provides a reasonable estimation of these values. The calculation also requires an established melting point of the mixture, which was not observed for mixtures with glycerol. Despite these limitations, the molar excess Gibbs energies were estimated, as shown in Table 2.

As shown in Table 2, where there is sufficient information to calculate the molar excess Gibbs energy, all of the mixtures examined here meet the requirement of a DES (that is, more negative than −1/3). For those where no melting point was observed in the DSC, we have included the highest melting point which would still meet the requirement of a DES.

None of the mixtures listed in Table 2 exhibited freezing or crystallization after >3 days at −20 °C. Therefore, it can be assumed that their melting points are below this, which means that they all meet the requirement of a deep eutectic solvent, based on the definition proposed by Like et al. Interestingly, after one week at −80 °C, all of the ethylene glycol-based mixtures had crystallized, but there was no evidence of crystallization in any of the glycerol-based mixtures. This further highlights the tendency of glycerol not to crystallize, making it an ideal candidate in the synthesis of DESs.

### 2.3. Viscosity

**Figure 2:**
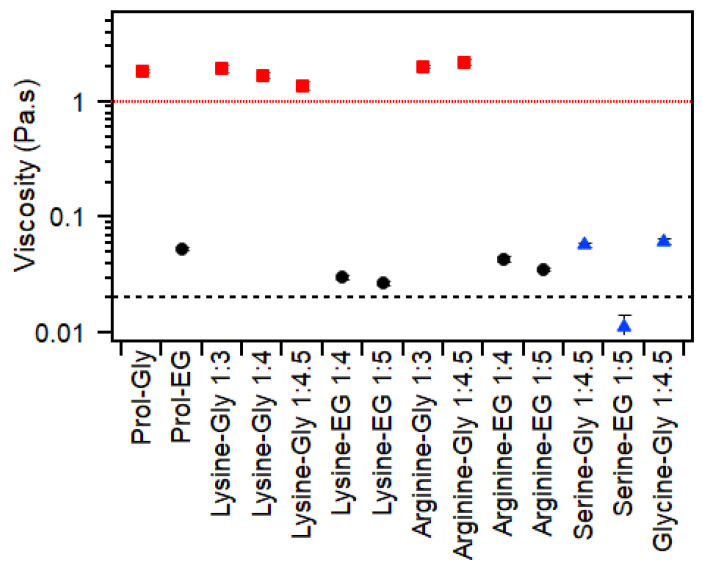
Viscosity of mixtures of amino acids with either glycerol (red squares) or ethylene glycol (black circles). The blue triangles show samples that had >20 wt% water. The red dotted line shows the value for pure glycerol, while the black dashed line shows the value for pure ethylene glycol. Data for proline-based samples were taken from a previous publication [3]. Error bars are based on the standard deviation of triplicate measurements and, in most cases, are smaller than the symbols.

The viscosity of these mixtures is highly dependent on the HBD, as shown in Figure 2. This has been previously reported for betaine-, choline chloride- and proline-based mixtures [3] and is attributed to the significant differences between the viscosity of glycerol (>1 Pa·s) and ethylene glycol (0.019 Pa·s) [35,36]. The viscosity of the glycerol-based mixtures was between 1 and 2 Pa·s, which is the same as that reported for betaine–glycerol (1:3, 1.2 Pa·s [3]) and slightly above that previously reported for choline chloride–glycerol (1:2, 0.2–0.5 Pa·s [3,37,38]) and choline chloride–urea (1:2, 0.9 Pa·s [3]). This is especially noteworthy as the mixtures studied here contained ~1 wt% water, while those examined previously reportedly contained < 1wt%, and it is well established that in these mixtures, the addition of even small amounts of water causes a significant decrease in viscosity [3]. The viscosity of the ethylene glycol mixtures ranged from 0.03 to 0.06 Pa·s, which is similar to that reported for choline chloride–ethylene glycol and betaine–ethylene glycol [3,39]. There were no clear links between the identity of the amino acid and changes to viscosity as the behavior was very much dominated by the HBD. The exception is seen for serine–glycerol (1:4.5), serine–ethylene glycol (1:5) and glycine–glycerol (1:4.5), all of which had greater than 20 wt% water, which is expected to significantly influence viscosity, and indeed, in the past literature, this water content reduced the viscosity of glycerol-based DESs by up to 50 times [3].

The above-mentioned study which described a range of amino acid-based DESs with glycerol reported that the viscosity of proline–glycerol (1:3) was 0.87 Pa·s (at 40 °C) and that of alanine–glycerol (1:3) was just 0.29 Pa·s (at 40 °C) [19]. These values are extremely low for glycerol-based mixtures, even at 40 °C, and further support the theory that there may be other components present which led to the formation of homogenous liquids, which was not observed in the current study. While that paper reported the formation of arginine and lysine mixtures, they did not report their viscosity.

### 2.4. Refractive Index

**Figure 3:**
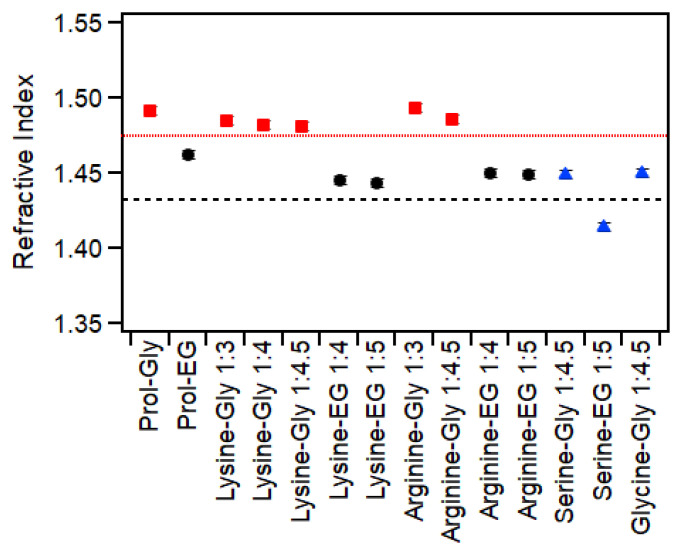
Refractive index of mixtures of amino acids with either glycerol (red squares) or ethylene glycol (black circles). The blue triangles show samples that had >20 wt% water. The red dotted line shows the value for pure glycerol, while the black dashed line shows the value for pure ethylene glycol. Data for proline-based samples were taken from a previous publication [3]. Repeated measurements demonstrated errors < 0.2%, which is smaller than the symbols.

As shown in Figure 3, the refractive index of all mixtures was significantly higher than that of water (1.333 [29]). The glycerol mixtures had higher values (closer to 1.5) than the ethylene glycol mixtures (close to 1.45), except for serine–glycerol (1:4.5), serine–ethylene glycol (1:5) and glycine–glycerol (1:4.5), all of which had greater than 20 wt% water, which has been shown to significantly decrease the refractive index of similar mixtures [3]. Glycerol has a refractive index of 1.4746, while for ethylene glycol, it is 1.4318 [29]; therefore, it is not surprising that the mixtures containing glycerol had higher values than those containing ethylene glycol, and the same pattern has been reported previously for other DESs, including choline chloride–urea which had a refractive index of 1.507 [3]. Refractive indices between 1.45 and 1.5 are comparable to those of organic solvents such as dimethyl sulfoxide, chloroform and toluene (1.4793, 1.4459 and 1.4941, respectively [29]). These results demonstrate that the refractive indices of these DESs can be reasonably predicted based on the components and that the identity of the amino acid has only a very minor affect. This could be used to fine-tune the refractive index of solvents for particular applications such as dynamic light scattering to allow better characterization of dispersed particles.

Molar refractivity is related to polarizability and can be calculated using the refractive index (Equation (2)) as has been carried out previously for ionic liquids [40],

(2) $$M_{R}=\;\frac{n^{2}-1\;}{n^{2}+1}\;\frac{MW}{\rho }$$

where n is refractive index, MW is molecular weight, and ρ is density. For DESs, the molecular weight is assumed to be an average of its components as shown in Equation (3),

(3) $${MW}_{DES}=\;\frac{\left({MW}_{a}\times N_{a}\right)+({MW}_{b}\times N_{b})\;}{N_{a}+N_{b}}$$

where ${MW}_{a}$ and $N_{a}$ are the molecular weight and number of moles of component a, and ${MW}_{b}$ and $N_{b}$ are the molecular weight and number of moles of component b. Therefore, in the case of proline–glycerol which contains 1 mol of proline and 3 of glycerol, Equation (3) becomes the following:

(4) $${MW}_{proline:glycerol}=\;\frac{\left(115\times 1\right)+(92\times 3)\;}{1+3}$$

Thus, the average molecular weight of the proline–glycerol DES is 98 g/mol.

Using these equations, the molar refractivity of proline–glycerol and proline–ethylene glycol is 28.8 m^3^/mol and 23.1 m^3^/mol, respectively. Only these two mixtures have published density data, and so analysis of the others is not possible. However, even from these two, it can be seen that the molecular refractivity of the mixture is closely related to that of the HBD, as the molar refractivities of glycerol and ethylene glycol (based on the above equations) are 27.0 m^3^/mol and 19.3 m^3^/mol, respectively.

### 2.5. Surface Tension

**Figure 4:**
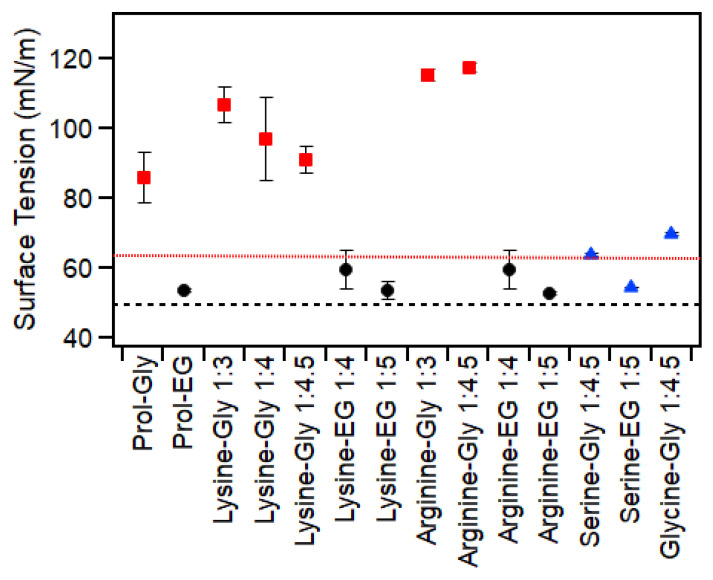
Surface tension of mixtures of amino acids with either glycerol (red squares) or ethylene glycol (black circles). The blue triangles show samples that had >20 wt% water. The red dotted line shows the value for pure glycerol, while the black dashed line shows the value for pure ethylene glycol. Data for proline-based samples were taken from a previous publication [3]. Error bars are based on standard deviation of triplicate measurements and, in some cases, were smaller than the symbols.

The surface tension of these DESs (Figure 4) was significantly influenced by the HBD, which is consistent with the previous literature [3]. As expected, mixtures containing ethylene glycol had significantly lower surface tensions (<60 mN/m) than those containing glycerol (>80 mN/m). In both cases, however, the surface tension of the mixture was higher than that of the pure HBD (63 mN/m for glycerol [41] and 48.6 mN/m for ethylene glycol [42]). This suggests that the presence of the amino acids adds to solvent cohesion and intermolecular interactions, leading to a higher surface tension at the air interface. This is further supported by the fact that at least for the glycerol-based mixtures, the order of surface tension was proline < lysine < arginine, which is the same order as their number of available hydrogen-bonding sites. This indicates that hydrogen bonding between the amino acids and glycerol leads to strong intermolecular forces and thus high surface tensions.

The results also suggest that the amino acids are in excess at the surface. This is supported by previous studies of amino acids in water which showed that amino acids strongly affect surface tension and are highly surface-active [43]. Another study has shown that the effect of an amino acid on surface tension in water depends on the identity of the amino acid and its relative hydrophobicity [44]. In that study, glutamine and serine caused an increase in surface tension, while valine, proline and methionine caused a decrease. While we cannot directly compare these results as the amino acid contents are vastly different (~25 wt% compared to 2 wt%), and the other component is not water, it does demonstrate the potential for amino acids to have surface activity. This, in turn, could lead to significantly different surface tensions compared to the pure HBD, as is observed here.

These measurements were taken at the air–liquid interface as quickly as possible (<30 s), as previous studies have demonstrated the significant uptake of water by these solvents at the interface [3]. Change in surface tension over time due to water uptake was further demonstrated for arginine–Gly (Figure S3). Other than a previous paper by our group examining mixtures of glycerol and ethylene glycol with proline, the surface tension of these mixtures has not been previously reported. Large error bars are attributed to the combined effects of (a) water uptake during measurement and (b) high viscosity of many of these samples.

Ultimately, the above results demonstrate that only a select few amino acids are capable of forming homogenous mixtures with glycerol and ethylene glycol at the ratios tested, as described in Table 3. The physical properties of the homogenous liquid samples are summarized in Table 1. It is obvious that the HBD (that is, glycerol or ethylene glycol) had a greater influence on the physical properties of these mixtures than the amino acid.

It is also clear that the formation of a homogeneous mixture (and by extension, a DES) cannot be predicted by simple parameters such as the size of the components, as the small amino acids (alanine and glycine) did not form stable solvents in any of the mixtures examined. It would appear that the presence of a charge (as in arginine and lysine) promotes DES formation, although this did not hold true for histidine. It may be that the ring structure of histidine interferes with solubilization and hydrogen bonding. This aspect is further complicated by the possibility of these amino acids existing as either non-ionic or zwitterionic molecules, with multiple pKa values (Table S3). As can be seen in Table S3, lysine and arginine have the highest isoelectric point (pI) of the amino acids studied here. This means that they are more likely to exist in a positively charged state near neutral pH, while the pI of the other amino acids was between 5 and 7, and therefore, they are more likely to be in an uncharged state which may reduce their solubility. The influence of this effect is further supported by measurements which showed that mixtures of glycerol with either lysine or arginine (1:4.5) when diluted with milliQ water had pH values of 11–12, while combinations with amino acids that did not form homogenous mixtures (e.g., glycine or serine) had pH values of 5–6. This suggests that deliberate alterations of the mixture’s pH, such as through the addition of an acid or base, may lead to the formation of a homogenous mixture, although it would be a complex combination of salts and solutes.

Amphiphilicity could not be linked to DES formation as proline readily formed homogenous mixtures, but none of the other amphiphilic amino acids did. However, proline has been noted to have an unusual structure, being the only amino acid tested to have a secondary amine [45].

Taken together, these results demonstrate that our understanding of the chemical properties and functional groups that promote DES formation is still in its infancy, and more rigorous and systematic studies will be required to establish clear design rules.

## 3. Materials and Methods

### 3.1. Synthesis

**Figure 5:**
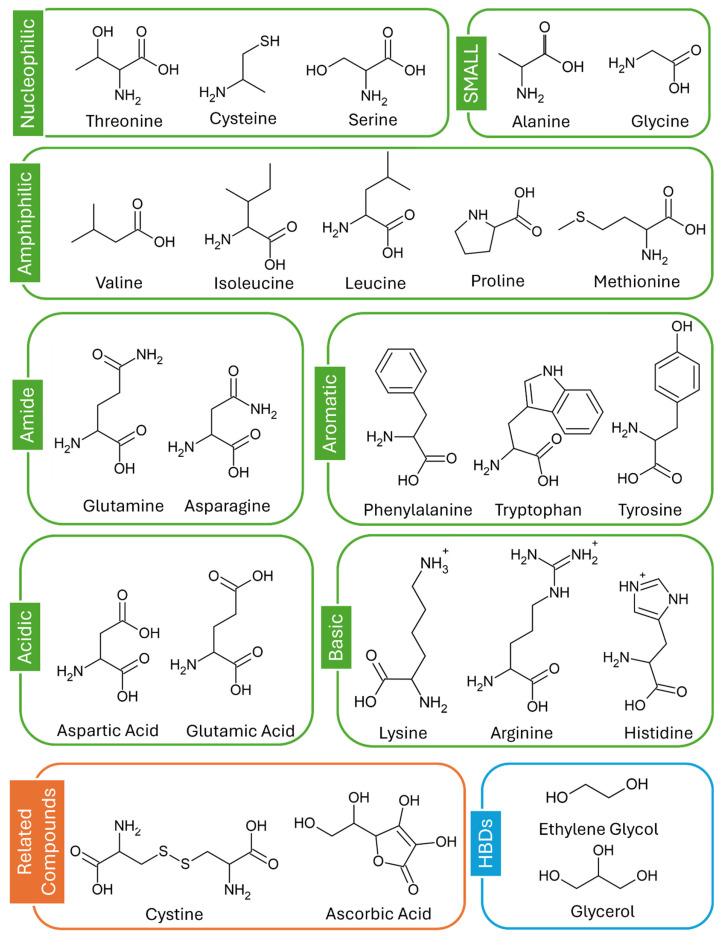
Chemicals used in this study.

The chemicals used in this study are shown in Figure 5. These were all purchased from Sigma and used as received: glycerol (Gly) (>99%), ethylene glycol (EG) (99%), L-proline (>99%), L-alanine (≥98), L-lysine (≥98), L-methionine (≥98), L-threonine (≥98), L-valine (≥98), L-tryptophan (≥98), L-tyrosine (≥98), L-phenylalanine (≥98), L-leucine (≥98), L-glutamic acid (≥99), L-glutamine (≥98), glycine (≥98), L-histidine (≥99), L-isoleucine (≥98), L-cystine (≥98), L-cysteine (≥97), L-asparagine (≥98), L-aspartic acid (≥98), L-ascorbic acid (≥98), L-arginine (≥98) and L-serine (≥99).

Each of the amino acids (or related compound) was combined with either glycerol or ethylene glycol at a ratio of 1:3 (amino acid–glycerol) or 1:4 (amino acid–ethylene glycol). Some mixtures were also tested at 1:4.5 (amino acid–glycerol) and 1:5 (amino acid–ethylene glycol). These ratios were based on previous studies reported in the literature [3,19]. The mixture was then heated to 90 °C for longer than 4 h, or until a homogeneous liquid formed. Each mixture was assessed, and if a homogenous liquid formed, further testing was carried out. Images of each of these mixtures are shown in Tables S1 and S2. For those that did not form a homogeneous mixture, water was added in 10 wt% increments, and the mixture was subjected to further heating/mixing as above. Mixtures that did not form a homogenous liquid at 50 wt% water were not investigated further. Mixtures that did form homogenous liquids were re-made with the addition of water at ~1 wt% increments. The results of this assessment are shown in Table 3.

### 3.2. Characterization

The thermal behavior of the mixtures which formed stable liquids was assessed using differential scanning calorimetry (DSC) with a 3+ StarE System (Mettler Toledo, Melbourne, Australia), calibrated with indium and zinc. Samples were loaded into aluminum pans (weighed using an MX5 microbalance (Mettler Toledo)) which were hermetically sealed. Samples were cycled three times between −150 °C and 30 °C, with a 5 min hold at each extreme. The resulting thermograms were analyzed for thermal events including freezing, melting and glass transitions using the StarE software (Mettler Toledo, v16.10). Because of kinetic effects, the thermal events are reported based on the heating rather than cooling cycles. The onset of melting of the glass phase is reported as the glass transition temperature, and the melting point is based on the onset of the highest-temperature melting event.

The viscosity of each of the mixtures was assessed using a temperature-controlled (25 °C) Discovery Hybrid Rheometer (HR20, TA Instruments, New Castle, DE, USA). Flow sweeps were collected between 0.1 and 1000 1/s. The reported viscosity is based on the average in the plateau region, usually at shear rates between 10 and 1000 1/s, as previously described [3].

Surface tension at the air–liquid interface was measured in triplicate using a Kibron Delta-8 Multi-Channel Tensiometer with 96-well plates. The instrument was calibrated before each session using MilliQ water and the probes cleaned using MilliQ water and a furnace between each measurement.

Refractive index was measured using a Rudolph Research J57 Automatic Refractometer (589.3 nm wavelength).

The approximate pH values of select samples were measured following substantial dilution in milliQ water using Hydrion pH strips.

## 4. Conclusions

This paper systematically tested mixtures of twenty amino acids and two related compounds with glycerol and ethylene glycol in an attempt to create a library of DESs. Contrary to the existing literature, most of these mixtures do not form homogenous liquids [19]. Only lysine and arginine (and as previously reported, proline) formed DESs with glycerol and ethylene glycol. With the addition of >20 wt% water, serine- and glycine-containing mixtures were also able to form homogenous liquids although these proved unstable.

The physical properties of these mixtures including thermal behavior, viscosity, refractive index and surface tension were characterized. These properties are most strongly influenced by the HBD. The identity of the amino acid had little effect on the viscosity and refractive index, but did affect melting point, glass transition temperature and surface tension.

This paper demonstrated a systematic and consistent method for characterization of potential DESs, as well as the measurement of important physical properties where possible. This is the first systematic report of the physical properties of these DES mixtures, which have strong potential biomedical applications including in fields such as cryopreservation.

Most importantly, the results demonstrate the need for more rigorous reporting of methods and data, in order to fill gaps in our current understanding. Therefore, future studies should focus on mixtures of amino acids with other HBDs and, as discussed above, the effect of pH on the formation of homogenous mixtures.

## Figures and Tables

**Table 1:** Mixtures characterized in this study, their water contents and physical properties.

Amino Acid	HBD	Ratio	Water (wt%)	Thermal Behavior (°C)	Viscosity (Pa·s)	RI (±0.003)	Surface Tension (mN/m)
Lysine	Glycerol	1:3	1.3	Glass: −74 ± 1	1.9 ± 0.2	1.485	106 ± 5
		1:4	1	Glass: −75 ± 1	1.7 ± 0.1	1.482	97 ± 10
		1:4.5	0.9	Glass: −76 ± 1	1.4 ± 0.1	1.481	91 ± 4
	Ethylene Glycol	1:4	1.1	Melt: −22 ± 1 Glass: −110 ± 1	0.03 ± 0.001	1.446	60 ± 6
		1:5	0.87	Melt: −21 ± 1 Glass: −112 ± 1	0.027 ± 0.001	1.443	54 ± 2
Arginine	Glycerol	1:3	10	Glass: −72 ± 3	2.0 ± 0.07	1.493	115 ± 2
		1:4.5	0.2	Glass: −74 ± 1	2.2 ± 0.1	1.486	117 ± 2
	Ethylene Glycol	1:4	0.3	Melt: −21 ± 1 Glass: −110 ± 1	0.043 ± 0.002	1.450	60 ± 5
		1:5	0.3	Melt: −2 ± 1 Glass: −111 ± 1	0.035 ± 0.001	1.449	52.8 ± 0.3
Serine	Glycerol	1:4.5	23	Glass: −95 ± 1	0.058 ± 0.002	1.449	63.8 ± 0.4
	Ethylene Glycol	1:5	29 ^A^	Glass: −122 ± 1	0.011 ± 0.003	1.414	54.5 ± 0.1
Glycine	Glycerol	1:4.5	21 ^A^	Glass: −94 ± 1	0.061 ± 0.004	1.450	69.6 ± 0.2

^A^ After 4 days at room temperature, these samples had evidence of precipitation and crystallization.

**Table 2:** Scaled molar excess Gibbs free energy of the eutectic, calculated using Equation (1). Values for those mixtures containing > 2 wt% water were not calculated. For those where no melting point was observed in the DSC, we have provided the highest melting point which would still meet the requirement of a DES according to Equation (1).

Mixture	Molar Ratio	Scaled Molar Excess Gibbs Free Energy	Maximum Melting Point to Meet DES Requirement (°C)
Lysine–Glycerol	1:3	No melting point in DSC	13
	1:4	No melting point in DSC	10
	1:4.5	No melting point in DSC	10
Lysine–Ethylene Glycol	1:4	−0.4	−15
	1:5	−0.4	−20
Arginine–Glycerol	1:4.5	No melting point in DSC	17
Arginine–Ethylene Glycol	1:4	−2.2	−4 *
	1:5	−1.8	−10 *

* enthalpy unknown, so these are very rough estimates.

**Table 3:** Results of mixing amino acids with either glycerol or ethylene glycol.

Amino Acid	HBD	Ratio	Result
L-Proline	Glycerol	1:3	Homogenous yellow/brown liquid
	Ethylene Glycol	1:4	Homogenous yellow liquid
L-Alanine	Glycerol	1:3	Precipitated crystals, even with >50 wt% water.
	Ethylene Glycol	1:4	Precipitated crystals, even with >50 wt% water.
L-Lysine	Glycerol	1:3	Homogenous yellow liquid
		1:4	Homogenous yellow liquid
		1:4.5	Homogenous yellow liquid
	Ethylene Glycol	1:4	Homogenous yellow liquid
		1:5	Homogenous yellow liquid
L-Methionine	Glycerol	1:3	Precipitated crystals, even with >50 wt% water.
	Ethylene Glycol	1:4	Precipitated crystals, even with >50 wt% water.
L-Threonine	Glycerol	1:3	Precipitated crystals, even with >50 wt% water.
	Ethylene Glycol	1:4	Precipitated crystals, even with >50 wt% water.
L-Valine	Glycerol	1:3	Precipitated crystals, even with >50 wt% water.
	Ethylene Glycol	1:4	Precipitated crystals, even with >50 wt% water.
L-Tryptophan	Glycerol	1:3	Precipitated crystals, even with >50 wt% water.
	Ethylene Glycol	1:4	Precipitated crystals, even with >50 wt% water.
L-Tyrosine	Glycerol	1:3	Precipitated crystals, even with >50 wt% water.
	Ethylene Glycol	1:4	Precipitated crystals, even with >50 wt% water.
L-Phenylalanine	Glycerol	1:3	Precipitated crystals, even with >50 wt% water.
	Ethylene Glycol	1:4	Precipitated crystals, even with >50 wt% water.
L-Leucine	Glycerol	1:3	Precipitated crystals, even with >50 wt% water.
	Ethylene Glycol	1:4	Precipitated crystals, even with >50 wt% water.
L-Glutamic Acid	Glycerol	1:3	Precipitated crystals, even with >50 wt% water.
	Ethylene Glycol	1:4	Precipitated crystals, even with >50 wt% water.
L-Glutamine	Glycerol	1:3	Precipitated crystals, even with >50 wt% water.
	Ethylene Glycol	1:4	Precipitated crystals, even with >50 wt% water.
Glycine	Glycerol	1:3	Precipitated crystals, even with >50 wt% water.
		1:4.5	Homogenous mixture at 21 wt% water *
	Ethylene Glycol	1:4	Precipitated crystals, even with >50 wt% water.
L-Histidine	Glycerol	1:3	Precipitated crystals, even with >50 wt% water.
	Ethylene Glycol	1:4	Precipitated crystals, even with >50 wt% water.
L-Isoleucine	Glycerol	1:3	Precipitated crystals, even with >50 wt% water.
	Ethylene Glycol	1:4	Precipitated crystals, even with >50 wt% water.
L-Cystine	Glycerol	1:3	Precipitated crystals, even with >50 wt% water.
	Ethylene Glycol	1:4	Precipitated crystals, even with >50 wt% water.
L-Cysteine	Glycerol	1:3	Precipitated crystals, even with >50 wt% water.
	Ethylene Glycol	1:4	Precipitated crystals, even with >50 wt% water.
L-Asparagine	Glycerol	1:3	Precipitated crystals, even with >50 wt% water.
	Ethylene Glycol	1:4	Precipitated crystals, even with >50 wt% water.
L-Aspartic Acid	Glycerol	1:3	Precipitated crystals, even with >50 wt% water.
	Ethylene Glycol	1:4	Precipitated crystals, even with >50 wt% water.
L-Ascorbic Acid	Glycerol	1:3	Precipitated crystals, even with >50 wt% water.
	Ethylene Glycol	1:4	Precipitated crystals, even with >50 wt% water.
L-Arginine	Glycerol	1:3	Formed a homogenous mixture at 10 wt% water
		1:4.5	Homogenous, slightly yellow liquid
	Ethylene Glycol	1:4	Homogenous, mostly clear liquid
		1:5	Homogenous, slightly yellow liquid
L-Serine	Glycerol	1:3	Homogenous mixture at 49 wt% water
		1:4.5	Homogenous mixture at 23 wt% water *
	Ethylene Glycol	1:4	Precipitated crystals, even with >50 wt% water.
		1:5	Homogenous mixture at 29 wt% water *

* These mixtures subsequently crystallized and precipitated, as discussed below.

## Data Availability

Data are contained within the article and Supplementary Materials.

## Supplementary Materials

The following supporting information can be downloaded at: https://www.mdpi.com/article/10.3390/molecules30040818/s1, Table S1: Photographs of amino acid:glycerol (1:3) mixtures after heating and stirring. All amino acids are L enantiomers unless otherwise stated. Table S2: Photographs of amino acid:ethylene glycol (1:4) mixtures after heating and stirring. All amino acids are L enantiomers unless otherwise stated. Figure S1: Example thermogram showing the complicated behaviour of some of the ethylene glycol based mixtures which had glass transitions, recrystallization and melting during heating. Figure S2: Thermogram of lysine-glycerol (1:4.5) showing a small freezing peak during cooling. Figure S3: Surface tension of arginine-Gly (1:4.5) measured over time. The blue line shows the surface tension of water (73 mN/m). Error bars are based on standard deviation of triplicate measurements. Table S3: pKa and pI values of amino acids used in this study. Data taken from, see ref [46].
