# Supplementary material for: Physico-Chemical Characterization of Amino Acid-Based Deep Eutectic Solvents
Source: Molecules. 2025 Feb 10;30(4):818. doi: 10.3390/molecules30040818 (PMC11858736; doi:10.3390/molecules30040818)
Supplement: Supplementary file 1 [file molecules-30-00818-s001.zip › molecules-3380396-supplementary.pdf]

Physico-Chemical Characterisation of Amino Acid-Based Deep Eutectic Solvents – Supplementary Information

Saffron J. Bryant,* Gary Bryant, Calum J. Drummond, Tamar L. Greaves

[1]School of Science, STEM College, RMIT University, Melbourne, VIC 3000, Australia.

Saffron.bryant@rmit.edu.au

Table S1. Photographs of amino acid:glycerol (1:3) mixtures after heating and stirring. All amino acids are L enantiomers unless otherwise stated.

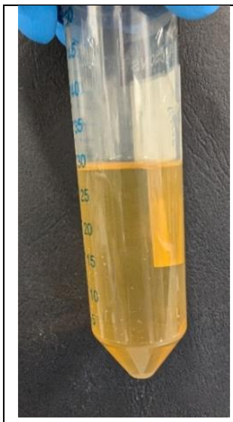	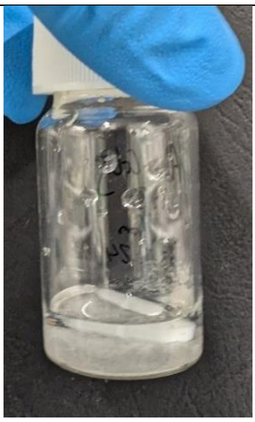	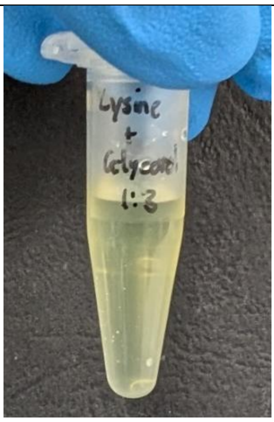	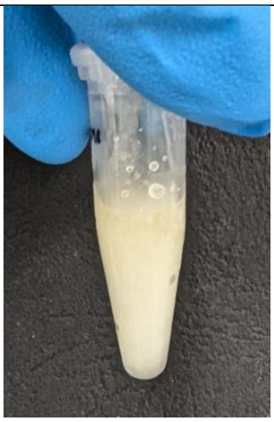
Proline	Alanine	Lysine	Methionine
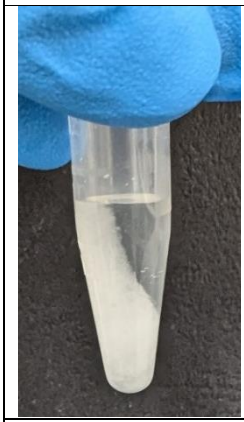	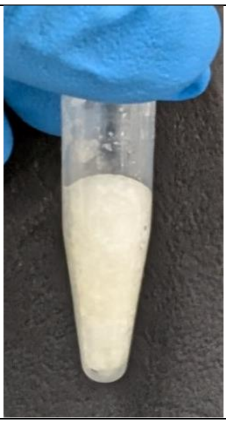	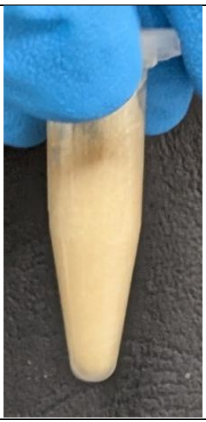	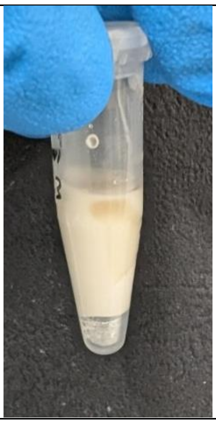
Threonine	Valine	Tryptophan	Tyrosine
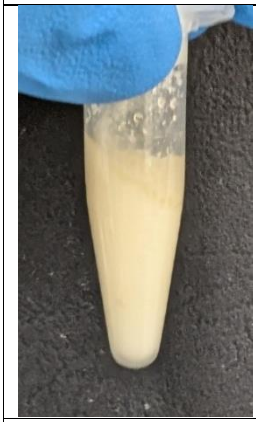	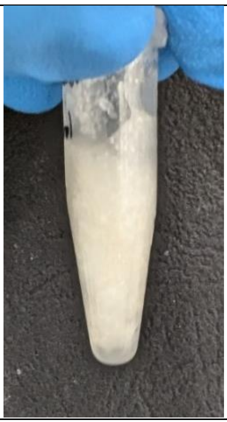	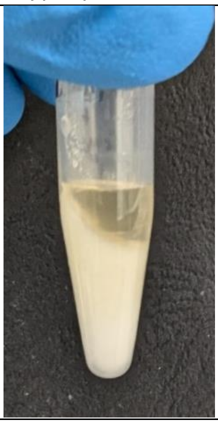	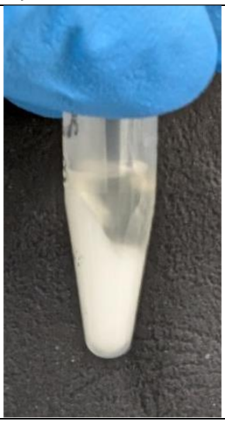
Phenylalanine	Leucine	Glutamic Acid	Glutamine

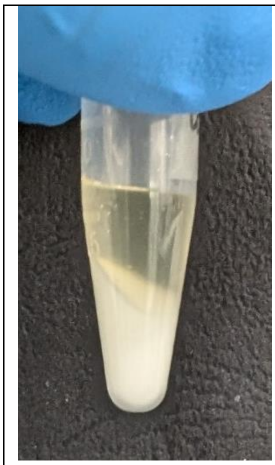	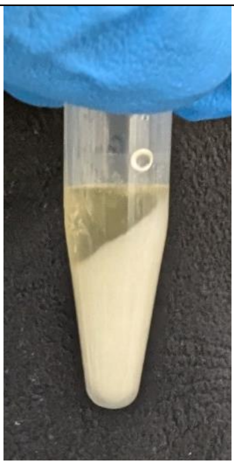	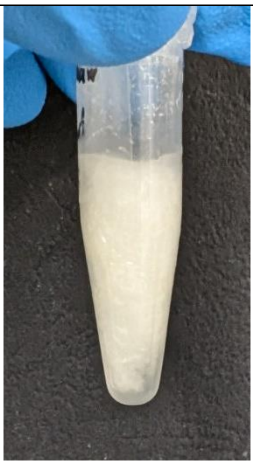	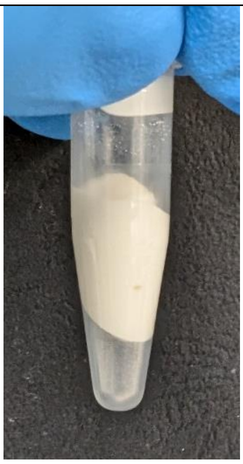
Glycine (non chiral)	Histidine	Isoleucine	Cystine
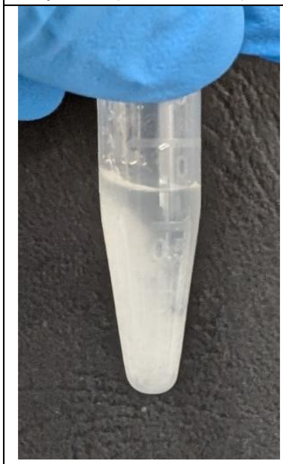	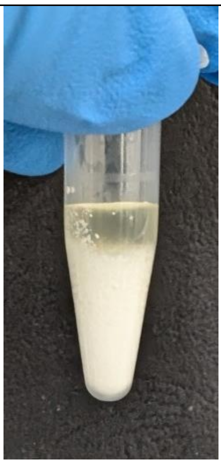	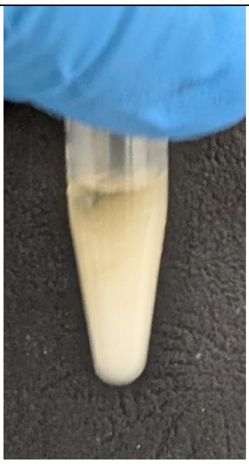	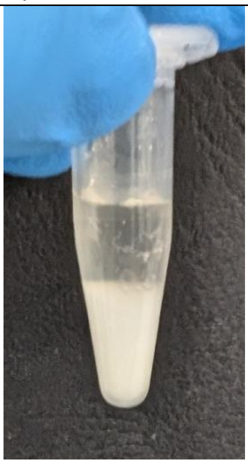
Cysteine	Asparagine	Aspartic Acid	Ascorbic Acid
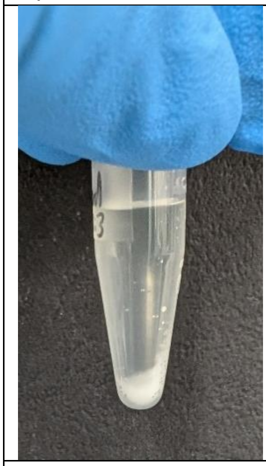	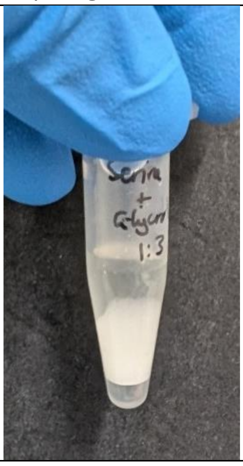		
Arginine	Serine		

Table S2. Photographs of amino acid:ethylene glycol (1:4) mixtures after heating and stirring. All amino acids are L enantiomers unless otherwise stated.

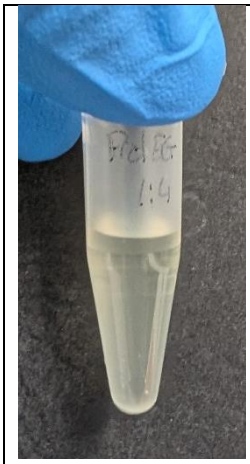	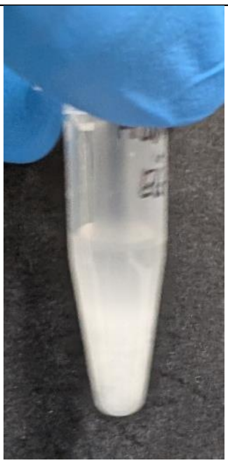	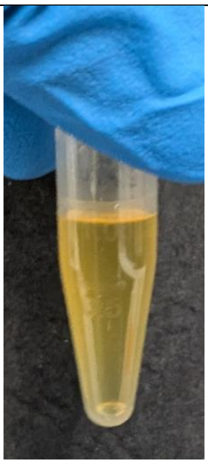	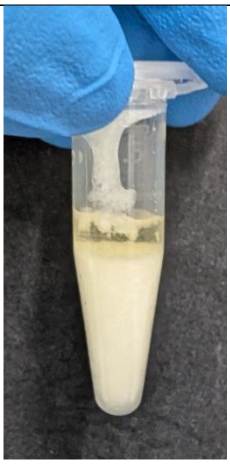
Proline	Alanine	Lysine	Methionine
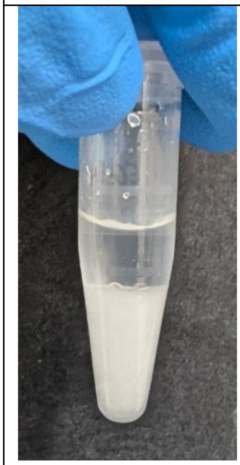	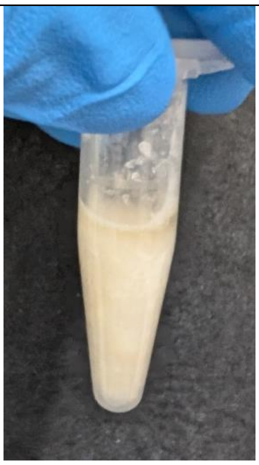	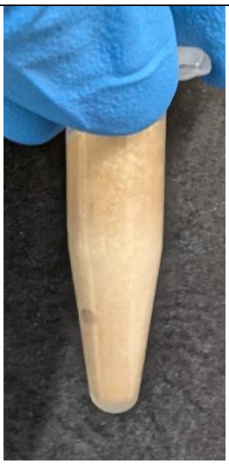	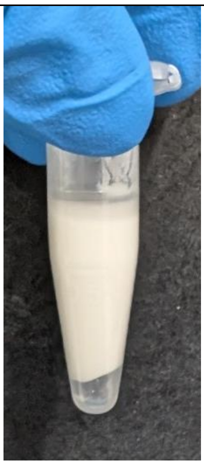
Threonine	Valine	Tryptophan	Tyrosine
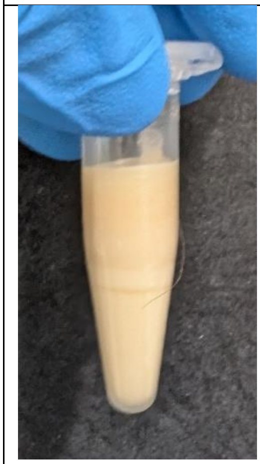	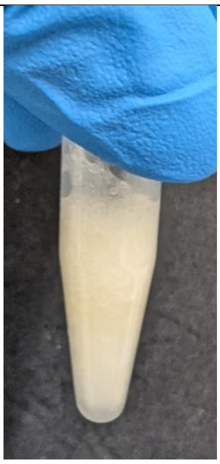	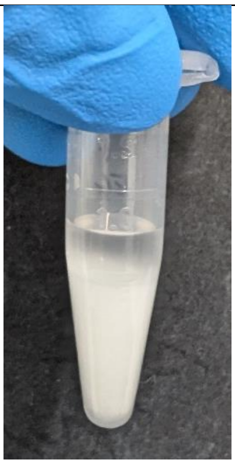	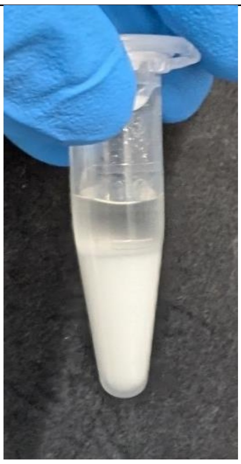

Phenylalanine	Leucine	Glutamic Acid	Glutamine
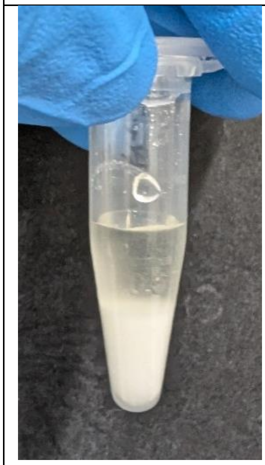	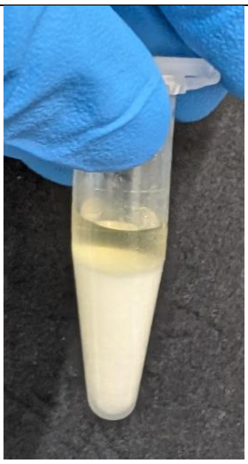	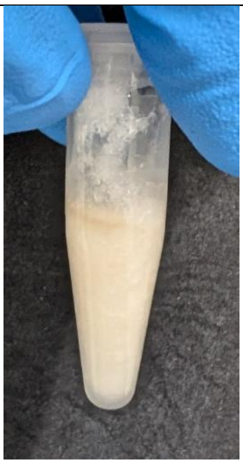	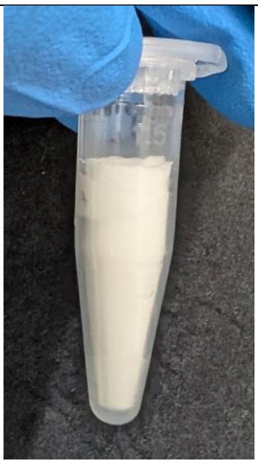
Glycine (non chiral)	Histidine	Isoleucine	Cystine
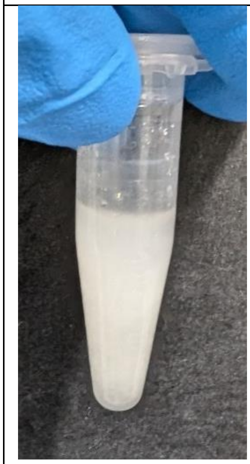	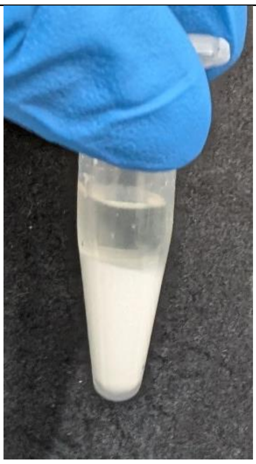	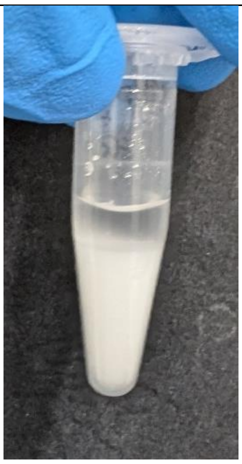	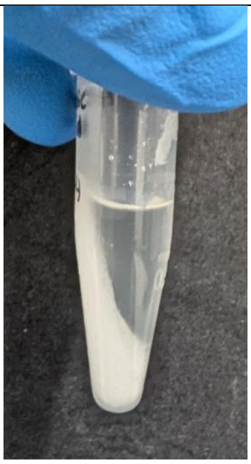
Cysteine	Asparagine	Aspartic Acid	Ascorbic Acid
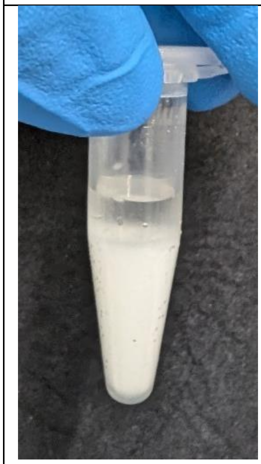	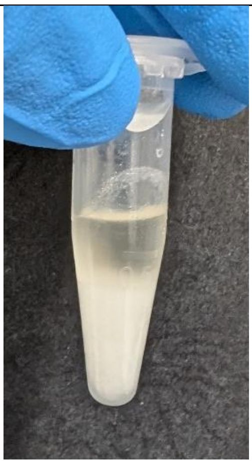		
Arginine	Serine		

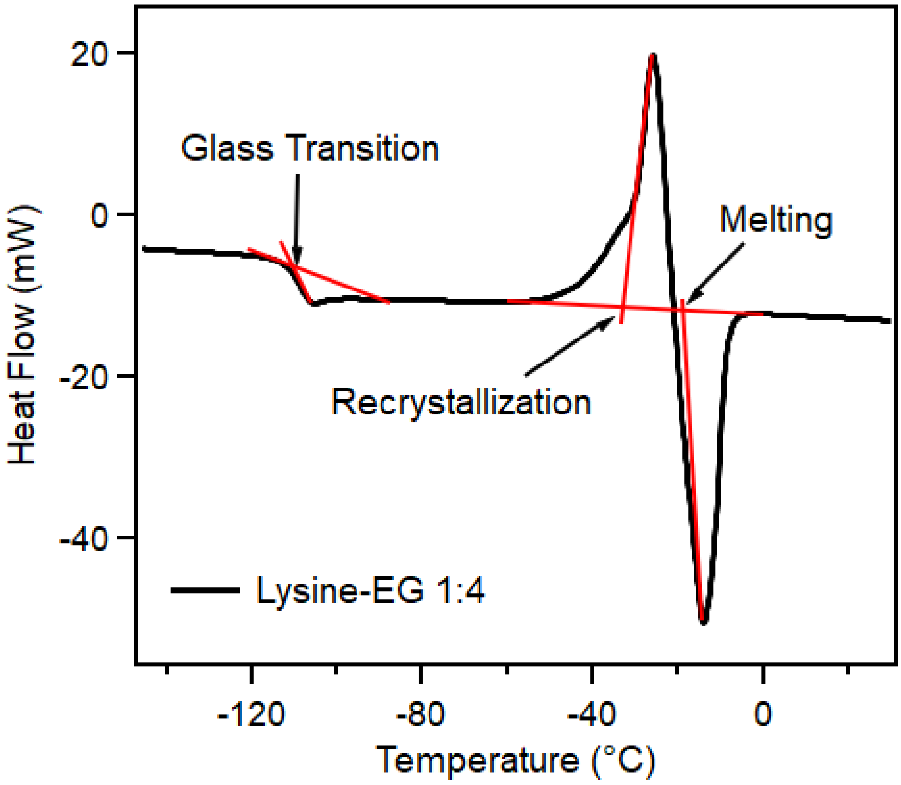

Figure S1. Example thermogram showing the complicated behaviour of some of the ethylene glycol based mixtures which had glass transitions, recrystallization and melting during heating.

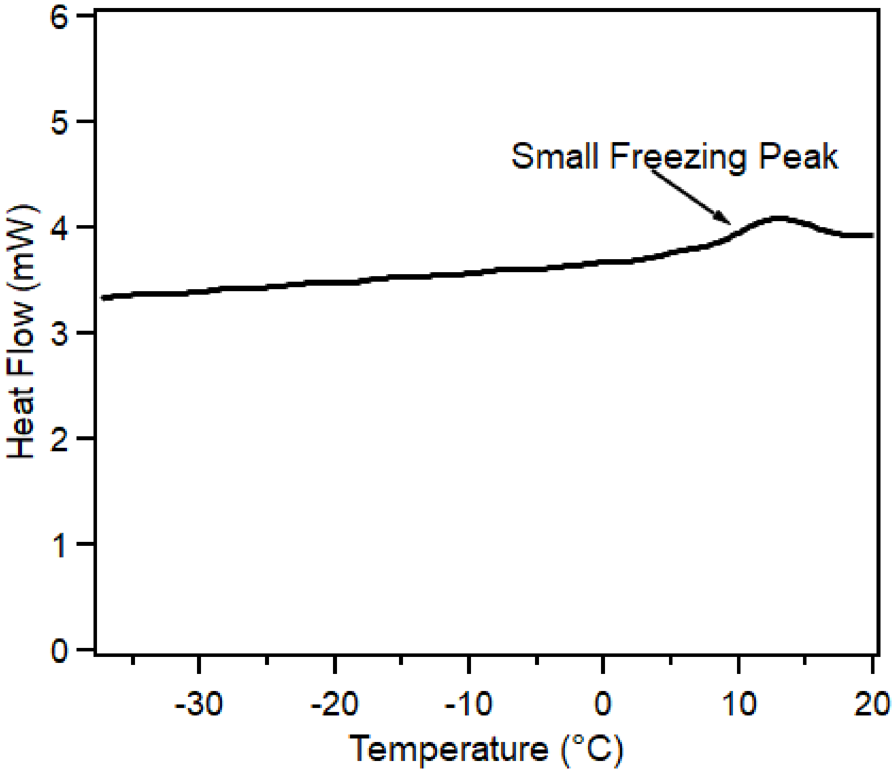

Figure S2. Thermogram of lysine-glycerol (1:4.5) showing a small freezing peak during cooling.

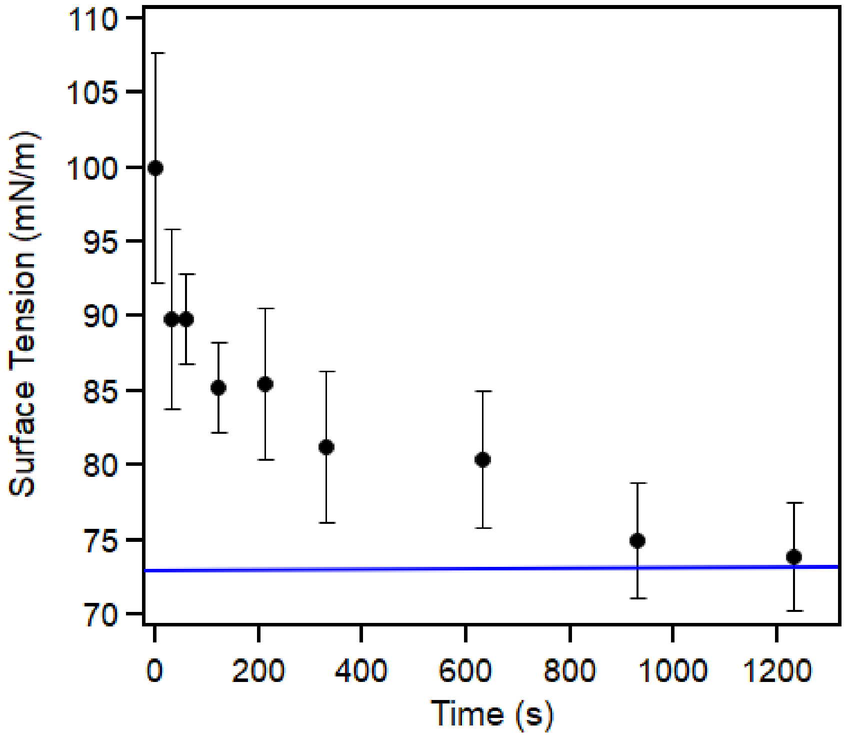

Figure S3. Surface tension of arginine-Gly (1:4.5) measured over time. The blue line shows the surface tension of water (73 mN/m). Error bars are based on standard deviation of triplicate measurements.

Table S3. pKa and pI values of amino acids used in this study. Data taken from [1].

Amino Acid	pKa1	pKa2	pKa3	pI
Glycine	2.34	9.60	-	5.97
Alanine	2.34	9.69	-	6.00
Valine	2.32	9.62	-	5.96
Leucine	2.36	9.60	-	5.98
Isoleucine	2.36	9.60	-	6.02
Methionine	2.28	9.21	-	5.74
Proline	1.99	10.60	-	6.30
Phenylalanine	1.83	9.13	-	5.48
Tryptophan	2.83	9.39	-	5.89
Asparagine	2.02	8.80	-	5.41
Glutamine	2.17	9.13	-	5.65
Serine	2.21	9.15	-	5.68
Threonine	2.09	9.10	-	5.60
Tyrosine	2.20	9.11	-	5.66
Cysteine	1.96	10.28	8.18	5.07
Aspartic acid	1.88	9.60	3.65	2.77
Glutamic acid	2.19	9.67	4.25	3.22
Lysine	2.18	8.95	10.53	9.74
Arginine	2.17	9.04	12.48	10.76
Histidine	1.82	9.17	6.00	7.59

References

(1) Francis A. Carey, R. M. G. Amino acids, peptides and proteins. In *Organic Chemistry*, 8 ed.; McGraw-Hill, 2011; p 1247.
